# Evaluation of immune protection against *Toxoplasma gondii* infection in mice induced by a multi-antigenic DNA vaccine containing TgGRA24, TgGRA25 and TgMIC6

**DOI:** 10.1051/parasite/2019050

**Published:** 2019-09-19

**Authors:** Xiao-Pei Xu, Wen-Ge Liu, Qian-Ming Xu, Xing-Quan Zhu, Jia Chen

**Affiliations:** 1 State Key Laboratory of Veterinary Etiological Biology, Key Laboratory of Veterinary Parasitology of Gansu Province, Lanzhou Veterinary Research Institute, Chinese Academy of Agricultural Sciences Lanzhou Gansu Province 730046 PR China; 2 College of Animal Science and Technology, Anhui Agricultural University Hefei Anhui Province 230036 PR China; 3 Ningbo University School of Medicine Ningbo Zhejiang Province 315211 PR China

**Keywords:** *Toxoplasma gondii*, DNA vaccine, Dense granule protein 24, Dense granule protein 25, Microneme protein 6

## Abstract

*Toxoplasma gondii* infection is prevalent in humans and animals worldwide. In this study, recombinant eukaryotic expression plasmids (pVAX-GRA24, pVAX-GRA25 and pVAX-MIC6) were constructed, and then injected into Kunming mice intramuscularly, as cocktailed plasmids or as single-gene plasmids. We evaluated immune protective responses by detecting the titer of antibodies and cytokine production of IFN-γ, IL-2, IL-4, IL-10, IL-12 and IL-23, the percentages of the subclasses of T lymphocytes, as well as the records of the survival time and cyst decrement in the brain of the mouse model after challenge with the *T. gondii* RH and Pru strains, respectively. Compared with the control groups, antibody and cytokine production were significantly increased, while the survival times of mice in all immunized groups were also prolonged, and the number of *T. gondii* cysts in their brains were decreased significantly (29.03% for pVAX-GRA24; 40.88% for pVAX-GRA25; 37.70% for pVAX-MIC6; 48.06% for pVAX-GRA24 + pVAX-GRA25; and 55.37% for pVAX-GRA24 + pVAX-GRA25 + pVAX-MIC6). The mouse group immunized with the three-gene cocktail (TgGRA24 + TgGRA25 + TgMIC6) had better performance in each detection index than the mouse groups immunized with the two-gene cocktail of TgGRA24 + TgGRA25, which was better than that in the group immunized with the single gene vaccine of TgGRA24, TgMIC6 or TgGRA25. In conclusion, TgGRA24 or TgGRA25 may be good vaccine candidates against *T. gondii* infection, but the three-gene cocktail of TgGRA24, TgMIC6 and TgGRA25 may induce the strongest protective immunity. Further studies of multi-antigenic DNA vaccines or cocktailed vaccines against *T. gondii* infection are necessary.

## Abstract (fr)

L’infection à *Toxoplasma gondii* est répandue chez les humains et les animaux dans le monde entier. Dans cette étude, des plasmides d’expression eucaryotes recombinants (pVAX-GRA24, pVAX-GRA25 et pVAX-MIC6) ont été construits, puis injectés à des souris Kunming par voie intramusculaire, comme cocktails de plasmides ou comme plasmides à un seul gène. Nous avons évalué les réponses de protection immunitaire en détectant le titre des anticorps et la production de cytokines IFN-γ, IL-2, IL-4, IL-10, IL-12 et IL-23, les pourcentages des sous-classes des lymphocytes T, ainsi qu’en mesurant les temps de survie et de décrément des kystes dans le cerveau du modèle souris après challenge par les souches RH et Pru de *T. gondii*, respectivement. Comparativement aux groupes témoins, la production d’anticorps et de cytokines était significativement accrue, tandis que le temps de survie des souris de tous les groupes immunisés était également prolongé et que le nombre de kystes de *T. gondii* dans leur cerveau diminuait de manière significative (29,03 % pour pVAX-GRA24 ; 40,88 % pour pVAX-GRA25 ; 37,70 % pour pVAX-MIC6 ; 48,06 % pour pVAX-GRA24 + pVAX-GRA25 ; 55,37 % pour pVAX-GRA24 + pVAX-GRA25 + pVAX-MIC6). Le groupe de souris immunisées avec les cocktails à trois gènes (TgGRA24 + TgGRA25 + TgMIC6) présentait la meilleure performance dans chaque indice de détection par rapport aux groupes de souris immunisées avec des cocktails à deux gènes de TgGRA24 + TgGRA25, qui était supérieur à ceux immunisés avec les vaccins monogéniques TgGRA24, TgMIC6 ou TgGRA25. En conclusion, TgGRA24 ou TgGRA25 peuvent être de bons candidats au vaccin contre l’infection à *T. gondii*, mais un cocktail à trois gènes de TgGRA24, TgMIC6 et TgGRA25 peut induire la plus forte immunité protectrice. Des études supplémentaires sur les vaccins à ADN multi-antigéniques ou les vaccins en cocktail contre l’infection à *T. gondii* sont nécessaires.

## Introduction

*Toxoplasma gondii* is an obligate intracellular protozoan parasite, with worldwide distribution, and is able to infect almost all warm-blooded organisms, including humans [8, 30]. *T. gondii* infections threaten human healthy, leading to severe disease in immuno-compromized individuals and the developing fetus [16]. In livestock, *T. gondii* infection can cause abortion and neonatal loss, particularly in sheep and goats, resulting in considerable economic losses [9, 10]. Although drug treatment can control acute *T. gondii* infections, it does not eliminate chronic infection with the tissues cysts of *T. gondii* [7]. Thus, immunoprophylaxis is considered to be a high priority to control and prevent *T. gondii* infections in humans and animals [18]. To date, a live attenuated S48 strain (Toxovax) is the only licensed vaccine used for the prevention of abortion in sheep infected with *T. gondii*; however, this vaccine has not been commonly used due to its inadequate efficacy and the potential risk of its reversion to virulence [8, 32]. Thus, it is urgent to develop more effective, practical and safe vaccines against *T. gondii* infection.

DNA vaccines can be prepared simply at low cost, and are able to generate effective immune responses [13]. Numerous studies have focused on the use of plasmid DNA to elicit protective immunity against *T. gondii* infection in animal models, including the virulence factors of *T. gondii*, such as ROP18, ROP5, GRA15, GRA6, MIC6 and MIC8 [6, 20, 24, 35]. However, no DNA vaccine candidates have been identified to induce complete protective immunity against *T. gondii* infection, so the identification of potential novel vaccine candidates against *T. gondii* infection will be the crucial step toward significant progress in the development of *T. gondii* vaccines [32, 39]. GRA24 is a dense granule protein, which could enter into the host cell nucleus, and modulate host immune response by directly interacting with p38α MAP kinase in the host cell. In addition, TgGRA24 can regulate the expression of many chemokines, such as CXCL10/IP-10, CCL2/MCP-1, CXCL1/GROα and CCL5/RANTES. During the acute stage of *T. gondii*, these chemokines could control *T. gondii* burden in infected organs [2, 4, 15, 19]. TgGRA25 is a novel virulence factor of *T. gondii*, impacting chemokine secretion, such as CCL2, which plays an important role in immune response during *T. gondii* infection. Mice lacking CCL2 can easily be infected with *T. gondii* [26, 28]. TgMIC6 is a constituent of the MIC1/MIC4/MIC6 complex, which is a well-characterized virulence factor of *T. gondii*, and also a DNA vaccine candidate against *T. gondii* infection in mice models [24, 34, 37].

Due to their critical biological roles in the pathogenesis of *T. gondii* infection, TgGRA24 and TgGRA25 may be promising DNA vaccine candidates against this parasitic infection. However, no previous studies have evaluated the vaccine potential of these two dense granule proteins. The objectives of this study were to evaluate the immunogenicity of TgGRA24 and TgGRA25 after injection of the constructed eukaryotic plasmids, containing different multi-components or a single-gene plasmid, respectively, and to assess the protective effects of these DNA vaccines against acute and chronic *T. gondii* infection in mice models.

## Materials and methods

### Ethics

All animals were handled under specific pathogen-free conditions, with the supply of food and water *ad libitum*, according to the Good Animal Practice requirements of the Animal Ethics Procedures and Guidelines of the People’s Republic of China. This study was approved by the Animal Ethics Committee of Lanzhou Veterinary Research Institute, Chinese Academy of Agricultural Sciences.

### Mice and parasites

Specific pathogen-free grade female Kunming mice (6–8 weeks old) were purchased from Lanzhou University Laboratory Animal Center (Lanzhou, China).

Tachyzoites of the highly virulent *T. gondii* RH strain (Type I) were preserved in the Department of Parasitology, Lanzhou Veterinary Research Institute (LVRI), Chinese Academy of Agricultural Sciences, China. Cysts of the *T. gondii* Pru strain were obtained from the brains of Kunming mice one month after oral administration of 20 cysts.

### Construction of DNA vaccine plasmid

The mammalian expression vector pVAX I was used as a DNA vaccine vector. The coding sequence of TgGRA24 and TgGRA25 was amplified by PCR from tachyzoite cDNA of the *T. gondii* RH strain using specific primers (forward primer: 5′-**GGGGTACC**ATGCTCCAGATGGCACGATATA-3′; reverse primer: 5′-**GCTCTAGA**TTAATTACCCTTAGTGGGTGGTTT-3′), which were designed based on the TgGRA24 gene sequence of the *T. gondii* GT1 strain (TGGT1_230180, http://www.toxodb.org/toxo/), containing *Kpn*I and *Xba*I restriction sites (in bold). The PCR product was ligated into the pMD-18 T Vector (TaKaRa, China), generating pMD-GRA24. The GRA24 fragment was cleaved by *Kpn*I/*Xba*I from pMD-GRA24 and then subcloned into pVAX I (Invitrogen), which was cleaved by *Kpn*I/*Xba*I. Plasmid pVAX-GRA25 was prepared in this study as mentioned above, with specific primers (forward primer, 5′-**GGGGTACC**ATGAAGCGTTTCTGGTTGT-3′, and reverse primer, 5′-**GCTCTAGA**TCAGTTTCTATCGAATTCCG-3′), and *Kpn*I and *Xba*I recognition sites were introduced. The plasmid pVAX-MIC6 was kept in the Department of Parasitology, LVRI, The People's Republic of China [24].

### *In vitro* expression of recombinant plasmid

The recombinant plasmids pVAX-GRA24 and pVAX-GRA25 were transfected into 293T cells using Lipofectamine 2000 reagent (Invitrogen, USA), according to the manufacturer’s instructions, and expression was assayed by indirect immunofluorescence assay (IFA). After 48 h of transfection, the cells were fixed in 12-well culture well plate with cold acetone and permeabilized with PBS-0.1% Triton-X-100 (PBST) for 15 min at each step. The cells were incubated with goat anti-*T. gondii* tachyzoite polyclonal antibody at 37 °C for 1 h, followed by FITC-labeled donkey-anti-goat IgG (Proteintech Group Inc., Chicago, USA) antibody at the dilution rate of 1:100 in PBST at room temperature for 45 min. At each step, the wells were washed three times with PBST. The specific fluorescence was examined using a Zeiss Axioplan Fluorescence Microscope (Carl Zeiss, Germany). 293T cells transfected with empty pVAX I were kept as a negative control.

### Immunization and challenge

Eight groups (30 Kunming mice per group) were used for this study, consisting of five experimental and three control groups. Mice in the experimental groups were immunized three times (2-week intervals) with 100 μL (1 μg/μL) of pVAX-GRA24, pVAX-GRA25, pVAX-MIC6, pVAX-GRA24 + pVAX-GRA25 or pVAX-GRA24 + pVAX-GRA25 + pVAX-MIC6 plasmids by intramuscular injection into the quadriceps, respectively. Control groups included mice injected with 100 μL empty pVAX I vector (1 μg/μL), 1× PBS or blank control, respectively. Two weeks after the third booster vaccine dose, 10 mice from all groups were challenged intraperitoneally with 1 × 10^3^ tachyzoites of the virulent RH strain, and the survival periods were recorded daily until all mice were dead. Meanwhile, another 10 mice per group were challenged with a non-lethal dose of 20 cysts of the Pru strain. Then, 4 weeks after the challenge, the surviving mice were sacrificed *via* cervical dislocation, and the mean number of cysts per brain was determined by counting three samples of 10 μL aliquots of each homogenized brain under an optical microscope. Two weeks after the last immunization, a total of 10 mice per group were sacrificed and splenocytes were aseptically harvested for a lymphocyte proliferation assay (three mice), cytokine measurements (another three mice), and flow cytometric analysis (another three mice). Blood was collected from the tail vein prior to each immunization and challenge (at weeks 0, 2, 4 and 6), and sera were separated and stored at –20 °C until analysis for specific antibodies. Pre-immune (at week 0) serum samples were used as negative controls.

### Antibody analysis

Enzyme-linked immunosorbent assay (ELISA) was used to detect IgG at 0, 2, 4 and 6 weeks, and thus to detect IgG1 and IgG2a antibodies in serum at 2 weeks after the last immunization by using an SBA Clonotyping System-HRP Kit (Southern Biotech Co., Ltd, Birmingham, AL, USA), as described previously [6]. In brief, 100 μL of capture antibody (10 μg/mL; provided by the commercial Kit) were added into each well and incubated overnight at 4 °C. After washing with PBST (1× PBS and 0.05% Tween 20), the wells were blocked with 100 μL of 1.0% BSA/PBS for 1 h, followed by adding detected sera and incubated at room temperature for 1 h. Plates were then washed, and anti-mouse-IgG, IgG1 and IgG2a antibodies conjugated with horseradish peroxidase (HRP) (Sigma-Aldrich, USA) were added to each well and incubated at 37 °C for 60 min. After washing with PBST, immune complexes were visualized by incubating with 100 μL substrate solution (1.05% citrate substrate buffer, 1.5% ABTS, 0.03% H_2_O_2_ of pH 4.0) for 30 min. The absorbance was measured at 405 nm using an ELISA reader (Bio-TekEL × 800, USA). All samples obtained from three different mice were run in triplicate.

### Lymphocyte proliferation assayed by MTT

Two weeks after the immunization with the third booster dose, splenocytes were collected from three mice in each group, as described previously [5]. The erythrocytes were lyzed using erythrocyte lysis buffer (0.15 M NH_4_Cl, 1.0 M KHCO_3_, 0.1 M EDTA, pH 7.2). After washing, the splenocytes were re-suspended in DMEM medium supplemented with 10% fetal calf serum (FCS). Briefly, 3 × 10^6^ cells per well were cultured in 96-well Costar plates with addition of concanavalin A (ConA) (5 μg/mL; Sigma, Sangon, China), or medium alone (negative control) at 37 °C under 5% CO_2_ for 72 h. Thereafter, 10 μL of 3-(4,5-dimethylthiazol-2-yl)-2,5-diphenyltetrazolium bromide (MTT, 5 mg/mL, Sigma, Sangon, China) was added to each well, and incubated for 4 h and the proliferative activity was measured according to the method described by Bounous et al. [3]. The stimulation index (SI) was calculated using the formula OD_570 ConA_/OD_570 M_. All samples obtained from three different mice were run in triplicate independently.

### Flow cytometry assay

To analyze the percentages of CD4^+^ and CD8^+^ T lymphocytes, a total of 1 × 10^6^ cells/mL were incubated with surface markers PE-CD3, APC-CD4 and FITC-CD8 antibodies (eBiosience, USA) at 4 °C for 30 min in a dark place, and then fixed with FACScan buffer (PBS containing 1% FCS and 0.1% sodium azide), and 2% paraformaldehyde. The samples were analyzed for fluorescence profiles on a FACScan flow cytometer (BD Biosciences, USA). All samples obtained from three different mice were run in triplicate independently.

### Cytokine assay

Splenocytes from each group were co-cultured with ConA for positive control and medium alone for negative control in flat-bottom 96-well microtiter plates. Culture supernatants were harvested for IL-2, IL-4 and IL-23 assay at 24 h, for IL-10 activity at 72 h, and for gamma interferon (IFN-γ) and IL-12p70 activity at 96 h using commercial ELISA kits according to the manufacturer’s instructions (Biolegend, USA). All samples obtained from three different mice were run in triplicate independently.

### Statistical analysis

The statistical analyses were performed using GraphPad Prism 7.0 and SPSS17.0 Data Editor (SPSS, Inc., IL, USA). The differences in the data (e.g., antibody titer and cytokine production) for all groups were compared by one-way ANOVA. *p* < 0.05 was considered statistically significant. The Kaplan–Meier method was used for analysis of the survival time of the RH strain.

## Results

### Expression of pVAX-GRA24 and pVAX-GRA25 plasmids in 293T cell

**Figure 1:**
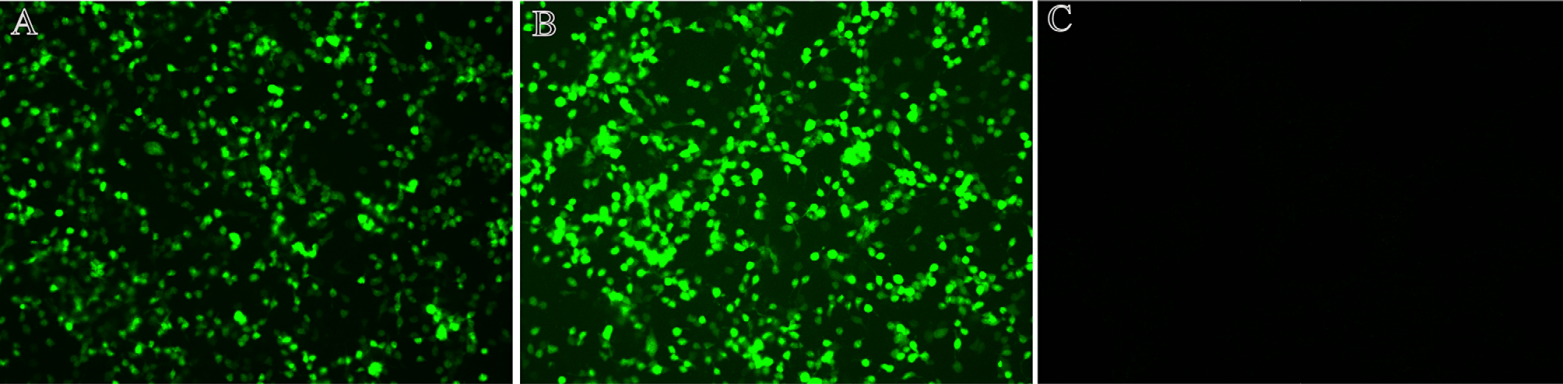
Immunofluorescence assay for the recombinant GRA24 and GRA25 protein expressed in 293T cells. 293T cells were transfected with pVAX-GRA24 (A) or pVAX-GRA25 (B) or empty pVAX I (C).

Expression of the recombinant plasmid was analyzed by indirect immunofluorescence (IFA). Specific green fluorescence was observed in 293T cells transfected with pVAX-GRA24 (Fig. 1A) and pVAX-GRA25 (Fig. 1B), whereas there was no fluorescence in cells transfected with empty pVAX I (Fig. 1C), indicating that the eukaryotic plasmid was successfully expressed in 293T cells.

### Humoral responses induced by DNA immunization

**Figure 2:**
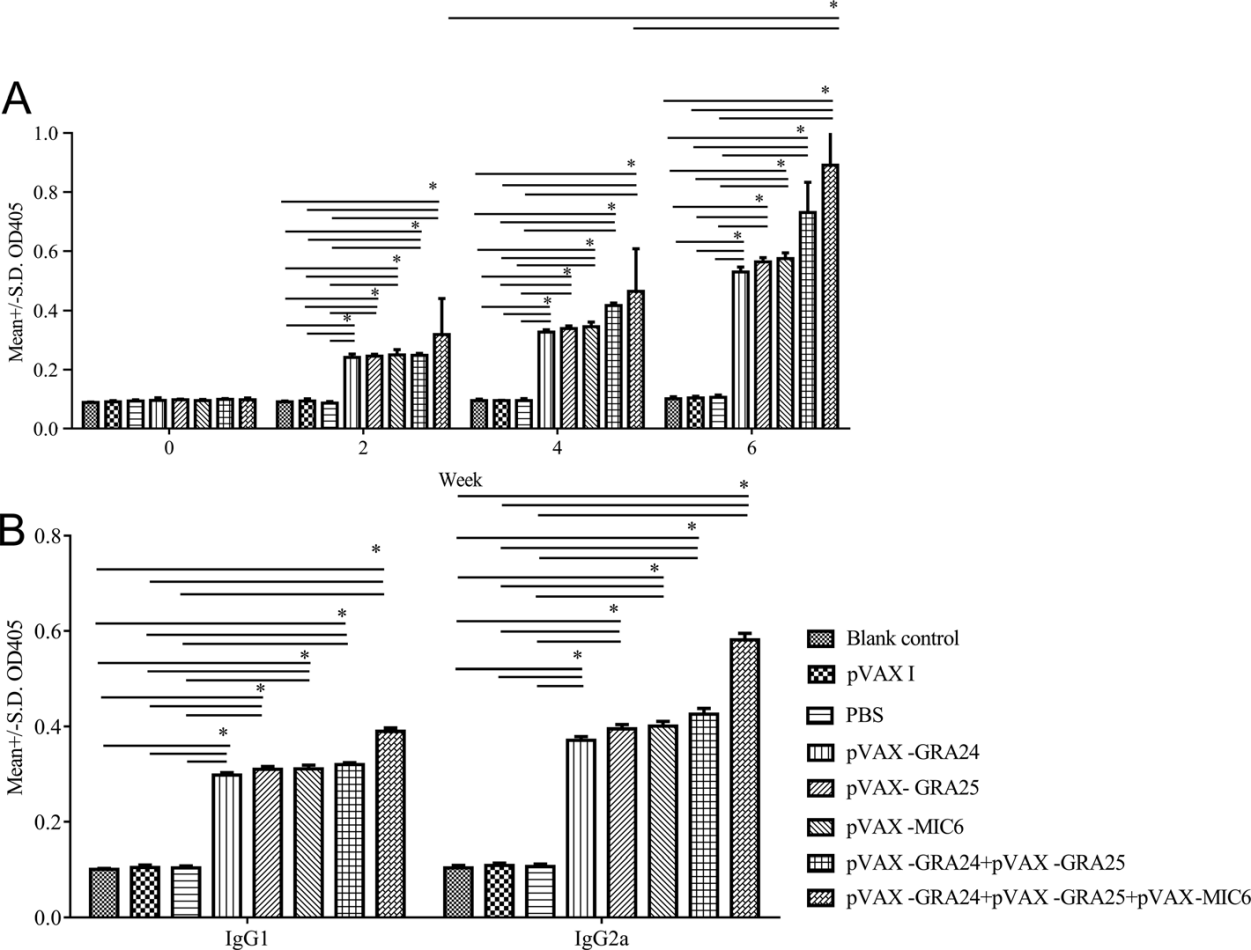
Detection of specific humoral immune responses. (A) Detection of total IgG antibody induced by experimental groups of mice on weeks 0, 2, 4, 6. (B) Detection of IgG1 and IgG2a antibodies in immunized mice groups 2 weeks after the last immunization. *Statistically significant difference (*p* < 0.05) between different immunized groups from the same measurement.

Significant antibody response was observed in all immunized mice. The highest antibodies titer was observed in mice immunized with the plasmid cocktail (pVAX-GRA24 + pVAX-GRA25 + pVAX-MIC6), as shown in Figure 2A. Also, boosting with pVAX-GRA24 and pVAX-GRA25 increased the IgG titer induced by DNA immunization with pVAX-GRA24 or pVAX-GRA25. The levels of IgG titer in the pVAX-GRA24, pVAX-GRA25 or pVAX-MIC6 groups were significantly higher (*p* < 0.05) than those in the three control groups. However, the levels of antibodies in the three control groups did not significantly increase with successive immunization (*p* > 0.05).

The levels of antibody subclass (IgG1 and IgG2a) isotypes were analyzed 2 weeks after the third immunization, showing a higher IgG2a to IgG1 ratio in experimental groups than that in the control groups, with the highest IgG2a to IgG1 ratio in groups of mice immunized with pVAX-GRA24 + pVAX-GRA25 + pVAX-MIC6 (Fig. 2B).

### Cellular responses induced by DNA immunization

**Figure 3:**
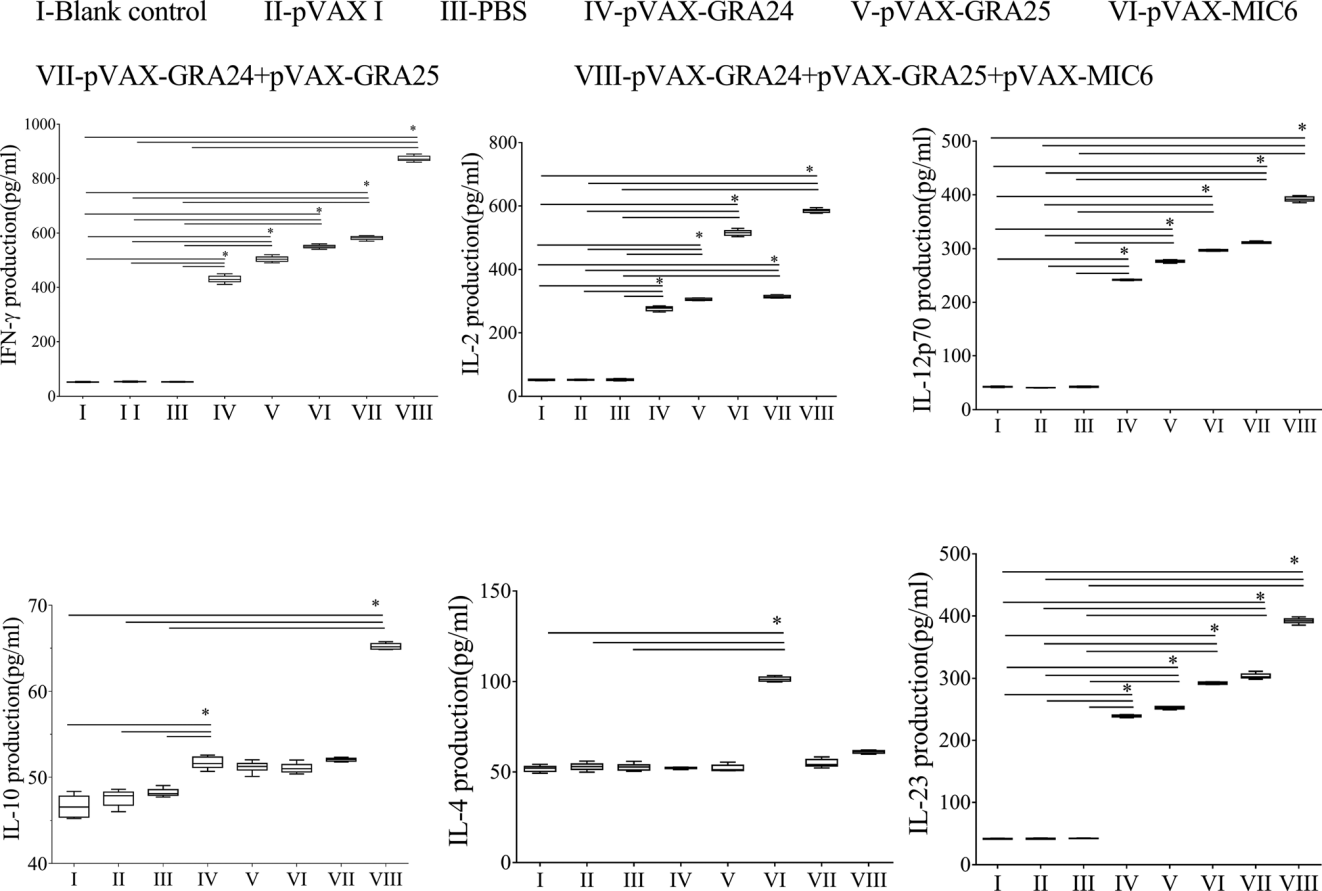
Cytokine production by splenocytes of mice immunized with single or multiple genes. *p < 0.05.

The results for humoral responses were similar to those for levels of cytokines from individual mice at 2 weeks after the third immunization. Co-injected mice induced the highest levels of IL-2, IFN-γ, IL-12 and IL-23, which were also significantly higher in experimental groups of mice immunized with a single antigen than in control groups. However, there was no significant difference in the levels of IL-4 and IL-10 between the groups of mice immunized with single and multiple-genes (*p* > 0.05) (Fig. 3).

### Splenocyte proliferation and the percentages of CD4^+^ and CD8^+^ of T lymphocytes

**Figure 4:**
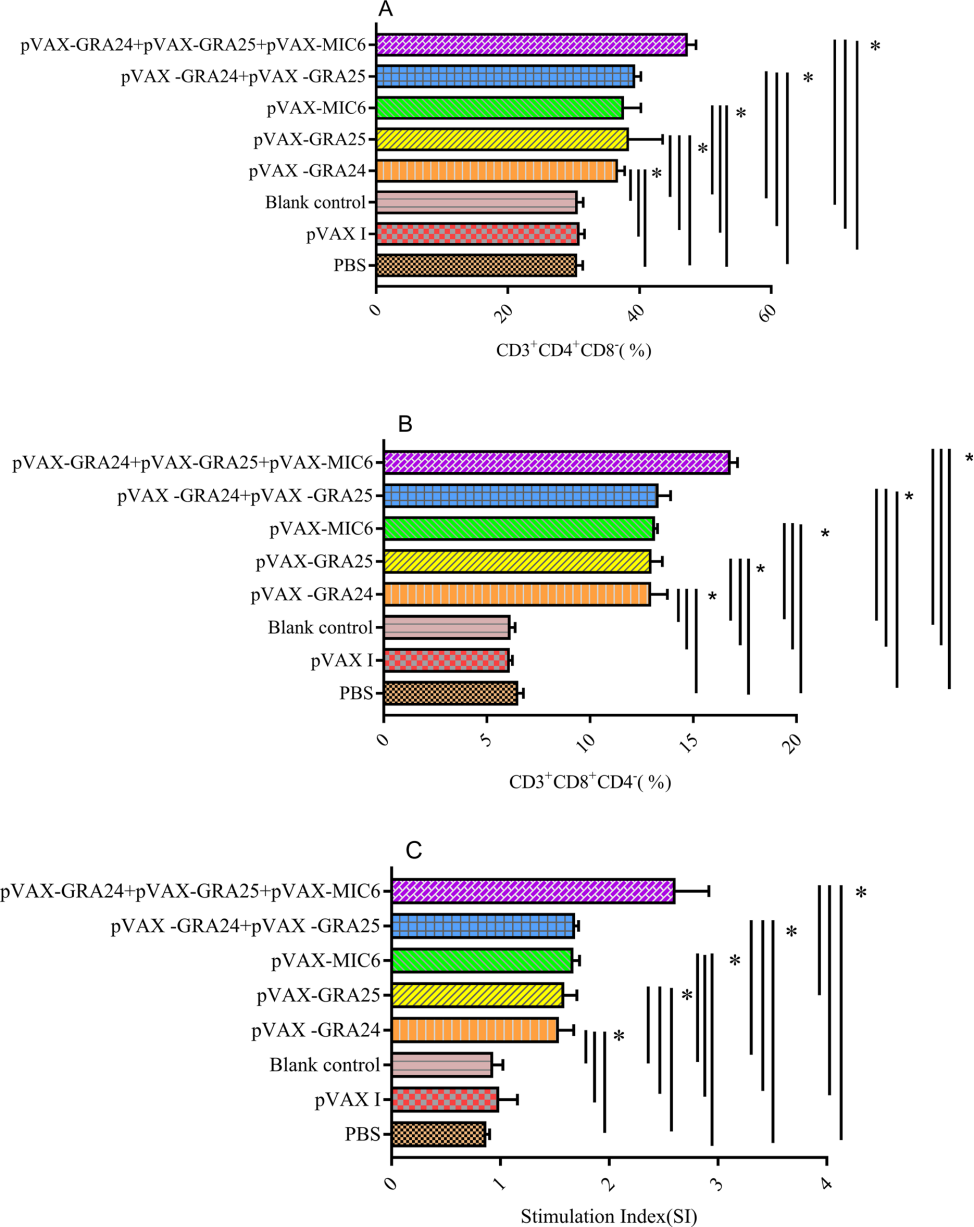
Splenocyte proliferation and the percentages of CD4^+^ and CD8^+^ of T cells in immunized and control mice. Determination of the percentages of CD4^+^ or CD8^+^ T cells in immunized and control mice (A, B). Lymphocyte proliferation stimulation index (SI) (C). *p < 0.05.

As shown in Figure 4A and B, the percentages of CD3^+^CD4^+^CD8^−^ and CD3^+^CD8^+^CD4^−^ T lymphocytes were significantly increased in the groups of mice immunized with pVAX-GRA24, pVAX-GRA25, pVAX-MIC6, pVAX-GRA24 + pVAX-GRA25, or pVAX-MIC6 + pVAX-GRA24 + pVAX-GRA25 compared with those in the three control groups (*p* < 0.05). In comparison, the groups of mice immunized with pVAX-GRA24 + pVAX-GRA25 showed higher percentages of CD3^+^CD4^+^CD8^−^ and CD3^+^CD8^+^CD4^−^ T lymphocytes than in the groups of mice immunized with a single-gene plasmid, but the highest percentages of CD4^+^ and CD8^+^ T lymphocytes were induced in the groups of mice immunized with pVAX-GRA24 + pVAX-GRA25 + pVAX-MIC6. Nevertheless, the three control groups showed no significant difference (*p* > 0.05).

Moreover, the proliferative response of lymphocytes was observed on spleen cells. Compared with the control groups, the SI was significantly higher in mice immunized with single or multiple gene vaccines (with the highest SI in co-injected mice) than in the three controls (Fig. 4C).

### Immuno-protection against lethal/non-lethal challenge

**Figure 5:**
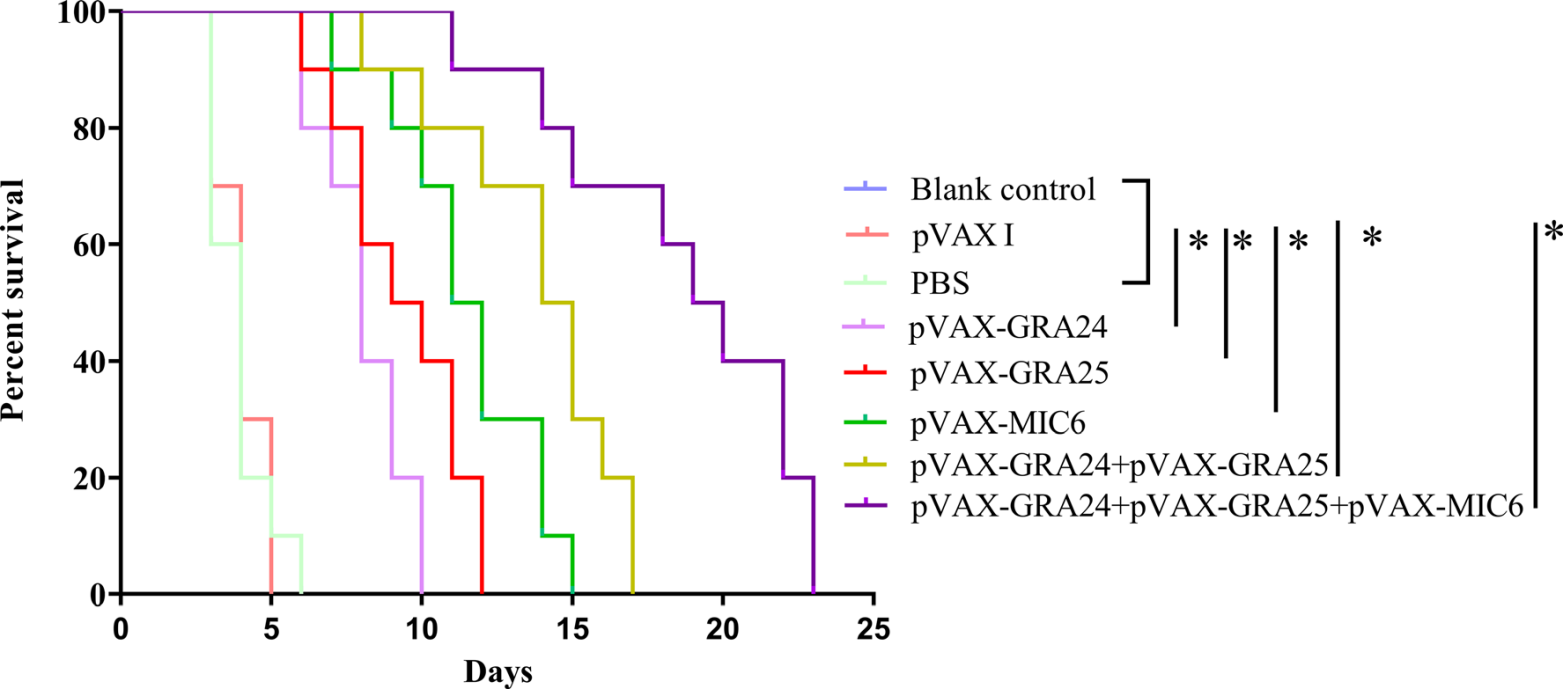
Survival rate of immunized Kunming mice followed by challenge with 1 × 10^3^ tachyzoites 2 weeks after the final immunization.

After challenge with 1 × 10^3^ tachyzoites of the virulent RH strain of *T. gondii*, mice immunized with pVAX-GRA24 (8.1 ± 0.5 days), pVAX-GRA25 (9.4 ± 0.7 days), pVAX-MIC6 (11.5 ± 0.8 days), pVAX-GRA24 + pVAX-GRA25 (13.8 ± 0.9 days) and with pVAX-GRA24 + pVAX-GRA25 + pVAX-MIC6 (18.7 ± 1.3 days) had a significantly longer survival time compared to the three control groups (Fig. 5) (*p* < 0.05). However, the mice in the three control groups died within 6 days after challenge (*p* > 0.05).

As shown in Table 1, the number of cysts in the mouse brain was reduced significantly in the pVAX-GRA24 (29.03%), pVAX-GRA25 (40.88%), pVAX-MIC6 (37.70%), pVAX-GRA24 + pVAX-GRA25 (48.06%) and pVAX-GRA24 + pVAX-GRA25 + pVAX-MIC6 (55.37%) groups, compared to the control group (*p* < 0.05). However, there was no apparent reduction of brain cysts among the three control groups (*p* > 0.05).

**Table 1:** Mean cyst burden per mouse brain 4 weeks after challenge with 20 cysts of *Toxoplasma gondii* Pru strain per mouse.

Group (*n* = 3)	No of brain cysts (Means ± *SD*)	Reduction (%)
Blank control	3011 ± 101*	–
pVAX I	2967 ± 110*	1.4
PBS	2980 ± 112*	1.03
pVAX-GRA24	2137 ± 99**	29.03
pVAX-GRA25	1780 ± 129**	40.88
pVAX-MIC6	1876 ± 113**	37.70
pVAX-GRA24 + pVAX-GRA25	1564 ± 102**	48.06
pVAX-GRA24 + pVAX-GRA25 + pVAX-MIC6	1334 ± 96**	55.37

* No statistically significant difference (*p* > 0.05) between different immunized groups from the same measurement.

** Statistically significant difference (*p* < 0.05).

## Discussion

Dense granules (GRAs) are known to maintain and form the nascent parasitophorous vacuole (PV) or a lipid-based intravacuolar network (IVN), or to be responsible for the uptake of nutrients from the host cell, and for intracellular parasite survival [22]. Due to their critical biological roles, some GRAs have been demonstrated to be effective DNA vaccine candidates, such as GRA1, GRA4, GRA6 and GRA7 [17, 27]. Here, the potential of TgGRA24 or TgGRA25 used as DNA immunization was evaluated in mouse models. The results suggest that DNA immunization with TgGRA24 or TgGRA25 could trigger effective protective immunity against acute and chronic *T. gondii* infection resulting from potent humoral and Th1-type cellular immune responses.

It is well known that a cocktail DNA vaccine could be used as a promising approach to elicit more potent protective immunity against acute and chronic toxoplasmosis, in contrast to that of a single antigen-based DNA vaccine [23, 31]. In the present study, two antigen-based DNA vaccines achieved a longer survival time and greater reduction in the parasite cyst burden than a single-gene plasmid. Furthermore, a cocktail of three antigens induced a prolonged survival time and reduction in the parasite cyst burden, which was greater than that of other vaccinations using multiple antigenic peptides [29, 38].

B cells, and thus IgG immunoglobulin-mediated immunity, are required to defend against acute *T. gondii* infection, and are involved in abilities of opsonizing *T. gondii* for phagocytosis or activating the classical complement pathway [6]. In this study, the levels of anti-*T. gondii* IgG antibodies were higher in the group immunized with the three-gene cocktail, followed by the two-gene cocktail, and single-gene vaccine, suggesting that these higher levels of antibodies may contribute to protection against subsequent acute infection with *T. gondii* tachyzoites, and to control brain cysts after challenge with the *T. gondii* Pru strain.

As the indicators for Th1-type cellular immune responses, cytokines including IL-2, IL-12 and IFN-γ were considered to be critical to the Th1 cell-mediated response against acute or chronic *T. gondii* infection [12, 21]. Also, higher titers of IgG subclass IgG2a isotype in sera favor Th1 responses [1, 36]. The IFN-γ cytokine is able to mediate killing intracellular *T. gondii via* innate or adaptive immune responses, which play a crucial role in resistance to *T. gondii* infection, in combination with IL-2 [25, 26]. Also, IL-12 could activate NK cells for the production of T-cell-independent mechanisms of resistance to infection, as well as drive development of Th1-type response through TLR stimulation of DC [25]. IL-23 is commonly considered to play a role in promoting the proliferation of T cells and IFN-γ generation, further in inducing memory T cells proliferation [21], and also its high levels were demonstrated to be induced by DNA vaccination against *T. gondii* infection in previous studies [5, 6]. In the present study, significant increases in IL-2, IL-12, IFN-γ, IL-23 and IgG2a were detected in all immunized groups, which suggests that all kinds of vaccines could induce vigorous Th1-based cellular immunity against *T. gondii.* Furthermore, the highest levels of cytokines in the group immunized with the three-gene cocktail indicate that cocktailed DNA vaccines could significantly enhance Th1 cell-mediated responses induced by a single DNA vaccine. The CD4^+^ T lymphocytes play an important role in the immune response against *T. gondii* infection, while in synergy with CD4^+^ T cells, CD8^+^ T lymphocytes are critical to prevent the development of brain cyst formation and tachyzoite spreading of *T. gondii* [11, 14]. In the present study, we found that the percentages of both CD8^+^ and CD4^+^ T cells were significantly increased in all immunized groups, while the highest increment was observed in mice immunized with the three-gene cocktail. The results of the present study are consistent with previous DNA vaccination studies based on multi-antigens, such as SAG1, ROP2, GRA2, ROP5 and GRA15 [6, 33].

The primary goal of the DNA vaccine construct is to provide further protective immunity against both acute and chronic toxoplasmosis caused by different *T. gondii* strains. In this study, we performed challenge models in mice, involving lethal infection with the wild-type (Type I) RH strain, and non-lethal infection with the low-virulent cyst-forming Pru strain (Type II). Our results show that DNA immunization with pVAX-GRA24 and pVAX-GRA25 induced a significantly longer survival time, and lower brain cyst number than that in a single DNA immunization, and also immunization with pVAX-GRA24 + pVAX-GRA25 + pVAX-MIC6 induced the best protective efficacy, which is also able to induce cross-protection between different genotypes of *T. gondii*.

In conclusion, the present study evaluated the immuno-protective effect of TgGRA24 and TgGRA25 in mice models, and demonstrated that both TgGRA24 and TgGRA25 are potential DNA vaccine candidates. Vaccination with cocktailed plasmids of TgGRA24, TgGRA25 and TgMIC6 induced better protective immunity against acute and chronic *T. gondii* infection in the mice model.
